# Modeling the environmental suitability of anthrax in Ghana and estimating populations at risk: Implications for vaccination and control

**DOI:** 10.1371/journal.pntd.0005885

**Published:** 2017-10-13

**Authors:** Ian T. Kracalik, Ernest Kenu, Evans Nsoh Ayamdooh, Emmanuel Allegye-Cudjoe, Paul Nokuma Polkuu, Joseph Asamoah Frimpong, Kofi Mensah Nyarko, William A. Bower, Rita Traxler, Jason K. Blackburn

**Affiliations:** 1 Spatial Epidemiology & Ecology Research Laboratory, Department of Geography, University of Florida, Gainesville, FL, United States of America; 2 Emerging Pathogens Institute, University of Florida, Gainesville, FL, United States of America; 3 Ghana Field Epidemiology and Laboratory Training Program, University of Ghana, Legon, Ghana; 4 Ministry of Food and Agriculture/ Veterinary Services Directorate, Tamale, Ghana; 5 Ministry of Food and Agriculture/ Veterinary Services Directorate, Accra, Ghana; 6 Noguchi Memorial Institute for Medical Research, University of Ghana, Legon, Ghana; 7 Bacterial Special Pathogens Branch, Division of High-Consequence Pathogens and Pathology, Centers for Disease Control and Prevention, Atlanta, GA, United States of America; University of California San Diego School of Medicine, UNITED STATES

## Abstract

Anthrax is hyper-endemic in West Africa. Despite the effectiveness of livestock vaccines in controlling anthrax, underreporting, logistics, and limited resources makes implementing vaccination campaigns difficult. To better understand the geographic limits of anthrax, elucidate environmental factors related to its occurrence, and identify human and livestock populations at risk, we developed predictive models of the environmental suitability of anthrax in Ghana. We obtained data on the location and date of livestock anthrax from veterinary and outbreak response records in Ghana during 2005–2016, as well as livestock vaccination registers and population estimates of characteristically high-risk groups. To predict the environmental suitability of anthrax, we used an ensemble of random forest (RF) models built using a combination of climatic and environmental factors. From 2005 through the first six months of 2016, there were 67 anthrax outbreaks (851 cases) in livestock; outbreaks showed a seasonal peak during February through April and primarily involved cattle. There was a median of 19,709 vaccine doses [range: 0–175 thousand] administered annually. Results from the RF model suggest a marked ecological divide separating the broad areas of environmental suitability in northern Ghana from the southern part of the country. Increasing alkaline soil pH was associated with a higher probability of anthrax occurrence. We estimated 2.2 (95% CI: 2.0, 2.5) million livestock and 805 (95% CI: 519, 890) thousand low income rural livestock keepers were located in anthrax risk areas. Based on our estimates, the current anthrax vaccination efforts in Ghana cover a fraction of the livestock potentially at risk, thus control efforts should be focused on improving vaccine coverage among high risk groups.

## Author summary

Anthrax is a soil-borne zoonotic disease found worldwide. In the West African nation of Ghana, anthrax outbreaks occur annually with a high burden to livestock keepers and their animals. To control anthrax in both humans and animals, annual livestock vaccination is recommended in endemic regions. However, in resource poor areas distributing and administering vaccine is difficult, in part, due to underreporting, logistical issues, limited resources, and an under appreciation of the geographic extent of anthrax risk zones. Our objective was to model high spatial resolution anthrax outbreak data, collected in Ghana, using a machine learning algorithm (random forest). To achieve this, we used a combination of climatic and environmental characteristics to predict the potential environmental suitability of anthrax, map its distribution, and identify livestock and human populations at risk. Results indicate a marked ecological divide separating the broad areas of environmental suitability in northern Ghana from the southern part of the country, which closely mirrors the ecotone transitions from southern tropical and deciduous forests to the northern Sudanian and Guinea Savanna. Based on our model prediction, we estimated >3 million combined ruminant livestock and low income livestock keepers are situated in anthrax risk zones. These findings suggest a low level of annual livestock vaccination coverage among high risk groups. Thus, integrating control strategies from both the veterinary and human health sectors are needed to improve surveillance and increase vaccine dissemination and adoption by rural livestock keepers in Ghana and the surrounding region.

## Introduction

Anthrax is a soil-borne, zoonotic disease found on nearly every continent (except Antarctica) that primarily infects herbivorous animals while secondarily infecting humans through the handling or ingestion of contaminated meat or animal by-products [1,2]. The geographic distribution of the disease appears to be limited by a combination of climatic (e.g. precipitation and temperature) and environmental (e.g. alkaline soil pH) conditions [3,4]. Under the appropriate ecological conditions, which remain poorly understood, the causative agent of anthrax, *Bacillus anthracis*, can survive for long-periods of time in the environment, perhaps years [1,4]. Although it has received much attention as a potential agent of bioterrorism, the World Health Organization (WHO) has listed anthrax as a neglected disease [5]. Poor livestock keepers and their animals often experience a disproportionate burden of anthrax in the hyper-endemic regions of Central Asia and West Africa [5,6]. Despite the effectiveness of regular animal vaccination and proper outbreak response following recommended guidelines in controlling anthrax in humans, underreporting of the disease often skews its true burden and geographic distribution making it difficult to implement adequate vaccination campaigns [1,7].

In Ghana, anthrax outbreaks have been reported annually in humans associated with contact with infected livestock and their contaminated animal by-products (e.g. meat or hides) [8]. Anthrax vaccine is manufactured locally by the Central Veterinary Laboratory in Pong-Tamale, Ghana and is fully subsidized by the government. Despite this, animal outbreaks are documented annually, and primarily affect cattle. Although both human and animal cases are reported, few human cases are linked to confirmed animal cases [9]. As a result, surveillance data alone provide limited information to efficiently plan prevention activities. Previous efforts to elucidate the environmental suitability of anthrax in Africa have been focused on southern countries, such as Zimbabwe [10], or national parks [11]. A recent study from West Africa also used a machine learning algorithm to map and model the distribution of anthrax and *B*. *anthracis* in Cameroon, Chad, and Nigeria, however, that effort was based on limited sample size and no comparable efforts have been carried out in Ghana [12].

To support Ghana’s national anthrax control and assessment, we our study had the following objectives: (1) model the environmental suitability of anthrax; (2) identify environmental and climatic factors associated with the occurrence of anthrax; (3) describe seasonal patterns; and (4) estimate populations at risk.

## Methods

### Ethics statement

This work was performed on nationally available data on anthrax outbreaks in livestock from the Ministry of Food and Agriculture in Ghana.

### Anthrax occurrence data

**Fig 1:**
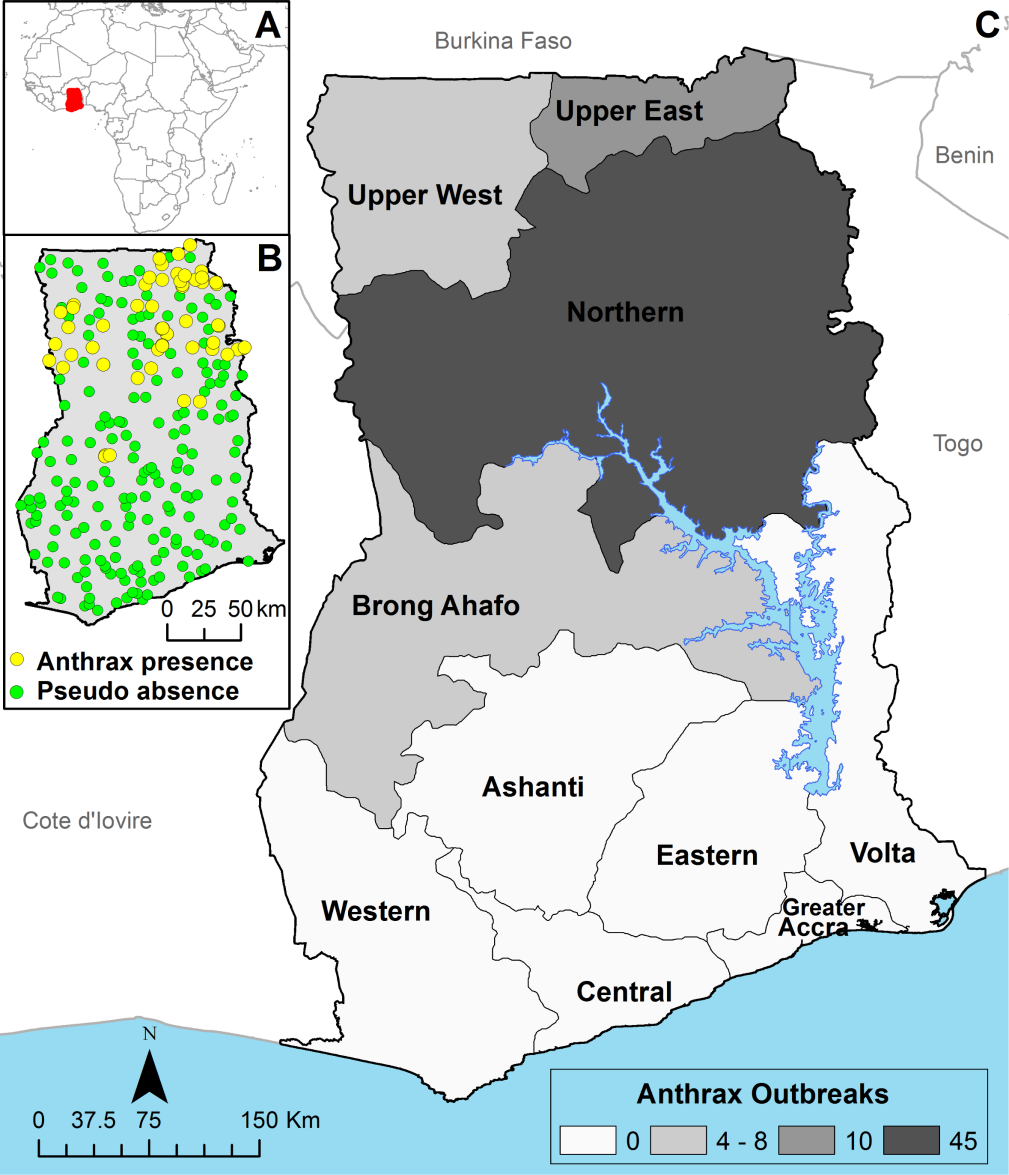
Spatial setting of Ghana in West Africa (inset A) and the geographic distribution of anthrax outbreaks (January 2005—June 2016) and generated pseudo absence data (inset B). The number of outbreaks by region in Ghana (inset C). Political boundary data were downloaded from www.gadm.org and all maps were produced in ArcGIS (www.esri.com; see Methods).

We constructed a GIS of livestock anthrax outbreaks using data collected by the Ghana Field Epidemiology and Laboratory Training Program (GFELTP) and the Ministry of Food and Agricultural Veterinary Services. (Fig 1). Outbreaks were mapped using GPS coordinates collected by field personnel responding to outbreaks or the center of the village where the outbreak occurred. We included data on outbreaks from 2005 through the first 6-months of 2016 included information on the geographic coordinates, date, livestock species, and number of individual animals infected (periodically recording mortality and survival status) for each outbreak. However, total livestock populations on affected properties was rarely reported. For this study, an outbreak was defined as any location with one or more anthrax cases in animals. We plotted the seasonality of anthrax outbreaks in relation to the average rainfall during 1991–2015 using data obtained from the Climate Change Knowledge Portal (http://sdwebx.worldbank.org/climateportal/index.cfm?page=country_historical_climate&ThisCCode=GHA). We also obtained livestock anthrax vaccine administration data during 2005–2015 from the World Animal health Information Database Interface (OIE; http://www.oie.int/animal-health-in-the-world/the-world-animal-health-information-system/data-after-2004-wahis-interface/). Mapping and spatial analysis was performed in Q-GIS version 2.14 (www.qgis.org) and the R statistical package (https://www.r-project.org/). Final maps were produced in ArcGIS version 10.3.1 (ESRI, Redlands, CA, USA).

### Environmental and climatic data

We used a combination of environmental and climatic variables at a spatial resolution of 30-arcseconds (approximately 1km x 1km) that followed, in part, recent studies in West Africa [13] and Central Asia [14] (Table 1). Five “bioclimatic” variables describing measures of temperature and precipitation were obtained from the WorldClim database (www.worldclim.org) [15]. WorldClim variables are interpolated monthly measurements recorded at weather stations located worldwide between 1950 and 2000. WorldClim produces bioclimatic variable grids to describe annual trends, seasonality, and ecological parameters such as temperature of the coldest and warmest quarters. We also used a combination of physical (sand content), chemical (soil pH), and taxonomic classifications of soil characteristics (cancerous vertisols and humults). Soil data were obtained from the SoilGrids1km database http://www.isric.org/explore/soilgrids) [16]. SoilGrid variables were created using spatial model predictions based on a global database of soil profiles and a combination of environmental covariates. Furthermore, we used two normalized difference vegetation index (NDVI) variables describing average conditions and the amplitude of vegetation greenness, which were obtained from the Trypanosomiasis and Land Use in Africa (TALA) research group (Oxford, United Kingdom) [17]. TALA variables were derived from temporal Fourier analysed (TFA) time series data of advanced very-high resolution radiometer (AVHRR) satellite measurements taken between 1992 and 1996 [17]. Mapped variables are shown in S1 Fig.

**Table 1:** Environmental and climatic variables used in the random forest models.

Environmental variable	Variable name	Data source	Reference
Elevation (m)	elevation	Worldclim	[6]
Mean annual temperature (°C)	bio 1	Worldclim	[6]
Annual temperature range (°C)	bio 7	Worldclim	[6]
Annual precipitation (mm)	bio 12	Worldclim	[6]
Precipitation: wettest month (mm)	bio 13	Worldclim	[6]
Precipitation driest month (mm)	bio 14	Worldclim	[6]
Average soil pH	soil pH	SoilGrids1km	[7]
Calcerous vertisols (% coverage)	vertisol	SoilGrids1km	[7]
Humult (% coverage)	humult	SoilGrids1km	[7]
Sand (% mass fraction)	sand	SoilGrids1km	[7]
Temporal Fourier mean NDVI	wd0114a0	TALA	[8]
Temporal Fourier NDVI annual amplitude	wd0114a1	TALA	[8]

### Data analysis

Random Forest (RF) modeling [18,19] was used to identify environmental characteristics associated with the occurrence of anthrax outbreaks using the ‘randomForest’ package for R. Previous studies have used this approach to map and model the distribution of *Anopheles spp*. mosquito vectors in Africa and Europe [20] and reservoirs of avian influenza [21]. RF modeling has been described and compared to other modeling approaches in detail elsewhere [18,22]. Briefly, RF is a non-parametric method derived from classification and regression trees that consists of a combination of trees built using randomly selected bootstrap samples of the training data (used to build the model), with the number of bootstrap samples equal to the number of trees (*ntrees*) selected. Each tree is split by randomly sampling a number of predictor variables to use (*mtry)* at each node and then choosing the best split. Model error estimates are obtained by internal splits of the training data (63.2% for model building) and then predicting the data not used to build a tree (out-of-bag or OOB) and aggregating these predictions for each ensemble of trees [18]. Since internal validation of the OOB data is performed, no external testing data is required to validate the model, but testing splits (external data withheld from the model) of the data are routinely utilized to assess model performance. Partial dependence plots and variable importance of RF models were assessed for covariates in the model.

We used an ensemble modeling approach that incorporated information from multiple random splits of our data into training (80%) and testing (20%) sets. Since our data consisted of presence only records of anthrax outbreaks, we generated pseudo-absence data from all available background data. Several studies have either relied on internal derivations of pseudo-absence in species distribution models [23] or user-defined generations such as in the modeling of the global distribution of dengue virus [24]. The required number of user-defined background pseudo-absence draws for every presence location is not standardized. It has been suggested that a 1:1 random draw of pseudo-absence to presence data in machine learning algorithms such as RF produces optimal results [25], although variations of this (2:1 or 3:1 draws) have been adopted successfully [24]. Similarly, pseudo-absence data creation has been shown to influence results; thus, research has recommended filtering pseudo-absence data from locations that are known to fall within suitable habitat or that occur within a defined proximity threshold [25,26].

We first filtered geo-located anthrax presence data in Ghana (n = 61) using a 5km x 5km proximity threshold in order to improve model performance and avoid overfitting [27]. We generated background pseudo-absence data (n = 200), from all available background [24], at a ratio of four absence points to every one filtered presence point (n = 50), restricting pseudo-absence data to exclude landscape within 5km of presence locations. We then generated 10 random draws each of 1:1, 2:1, and 3:1 pseudo-absence to presence data (30 total draws) with replacement. Each randomly generated pseudo-absence to presence draw (n = 30) was randomly divided into training and testing data splits to validate model performance. The final RF models were built using a *mtry =* 4 at each split and *ntrees* = 1000 with a combination of variables in which the ensemble list contributed to a mean decrease in accuracy >1%. The 30 individual RF models were then combined into an ensemble prediction at a spatial resolution of ~1km x 1km and scaled from 0 (low suitability) to 1 (high suitability); uncertainty in the model prediction was calculated by taking the range in the 95% confidence intervals of the ensemble model scaled from 0 (low uncertainty) to 1 (high uncertainty) following Deribe et al. [28].

The resulting output of our ensemble RF model represents the environmental suitability of anthrax in Ghana. To estimate the number of livestock and poor rural livestock keepers at risk in anthrax suitable areas, we dichotomized the modeled environmental suitability into a suitable versus not suitable prediction using a probability threshold that maximized sensitivity and specificity. We then overlaid a database of global livestock density at a spatial resolution of ~1km x 1km (http://www.livestock.geo-wiki.org/) [29] with the dichotomized anthrax prediction to estimate the livestock populations (cattle, sheep, goats, and swine) at risk. Livestock populations at risk were further stratified to estimate the population at risk within each of the livestock production zones of Ghana using the livestock production systems data version 5 (http://www.livestock.geo-wiki.org/) [29–31]. Furthermore, we estimated the number of low income rural livestock keepers at risk within each livestock production zone by overlaying the dichotomized anthrax suitable areas with estimates of the population of low income rural livestock keepers provided in Robinson et al. [31] and deriving the fraction of cells that were within our model prediction. Uncertainty in the populations at risk and 95% confidence intervals were calculated by using the 2.5% (lower) and 97.5% (upper) bounds of the ensemble RF model prediction [28].

Model performance and validation was conducted for each individual RF model and included the internal: OOB error classification, area under the receiver operating characteristics curve (AUC), sensitivity, and specificity. Additionally, we performed accuracy assessments on the external testing data, which consisted of thirty random subsets of 20% of the data sampled with replacement. Mean values and 95% confidence intervals were estimated for each accuracy metric. The AUC has been used extensively in species distribution modeling to measure the discriminatory performance of models [32]; an AUC value of 1 indicates a perfect discrimination while values of >0.9 are outstanding, 0.8–0.9 excellent, 0.7–0.8 acceptable, and <0.7 indicate poor discriminatory performance [28,33].

## Results

### Anthrax outbreaks

**Fig 2:**
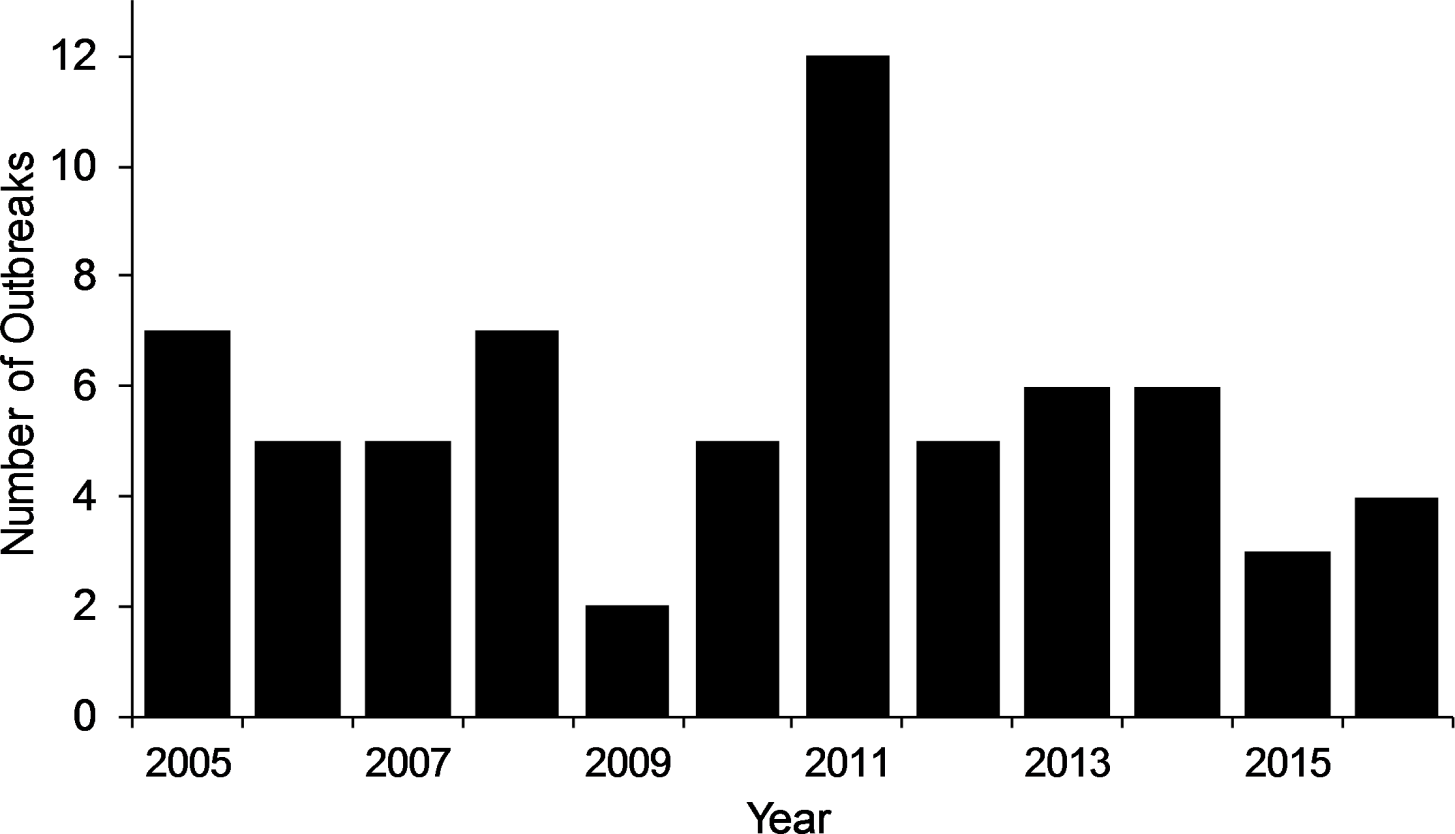
Annual number of livestock anthrax outbreaks in Ghana during January 2005- June 2016.

From 2005 through the first 6 months of 2016, there were 67 reported anthrax outbreaks in livestock (61 that were geo-located) (Fig 1). Nationally, there was a mean of 6 (95% CI: 4, 7) outbreaks per year with a peak in 2011 (n = 12) and lull in reporting in 2009 (n = 2) (Fig 2). The geography of outbreaks shows a higher frequency of anthrax in northern Ghana in the Upper East and Northern regions. Of the reported outbreaks, 4 (6%) were comprised of two or more livestock types. Domestic cattle were reported in 53% (35) of outbreaks, followed by sheep in 32% (21), goats in 11% (7), and swine in 5% (3). During 2005–2016, cattle anthrax cases were reported every year except in 2009. Sheep cases were ubiquitous annually and were characterized by a large number of deaths in 2012, the same year there was also a large number of swine cases (n = 500) (Table 2).

**Table 2:** Cases of anthrax by livestock type in Ghana, 2005–2016.

Year	Cattle	Sheep	Goats	Swine
2005	27	1	0	0
2006	17	15	0	0
2007	15	9	3	0
2008	21	6	11	0
2009	0	2	0	0
2010	2	1	0	0
2011	13	7	0	3
2012	6	66	1	500
2013	50	14	0	0
2014	3	1	0	1
2015	26	1	0	0
2016	4	6	19	0
**Totals**	**184**	**129**	**34**	**504**

**Fig 3:**
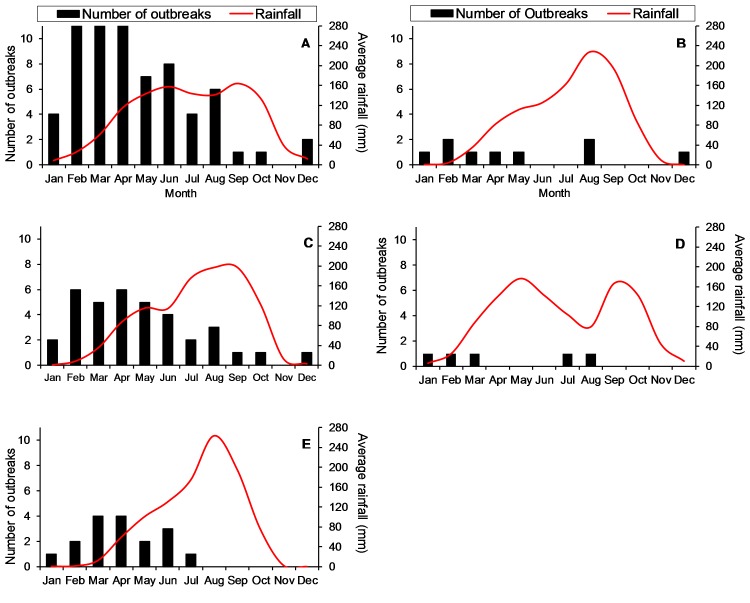
Seasonal distribution of anthrax outbreaks (black bars) during 2005–2016 with average rainfall totals (red line) in Ghana nationally (A) and by region: Upper West (B), Northern (C), Brong Ahafo (D), and Upper East (E). Anthrax outbreaks by Region.

The seasonality of anthrax outbreaks nationally and regionally are illustrated in Fig 3. Nationally, outbreaks were reported, on average, across seasons and in every month (except November). There was an increase in outbreaks in the late winter and early spring months, with February through April having the highest reported number of outbreaks. On average, there outbreaks appeared to occur in the dry season before the onset of the rains.

### Livestock vaccination

**Fig 4:**
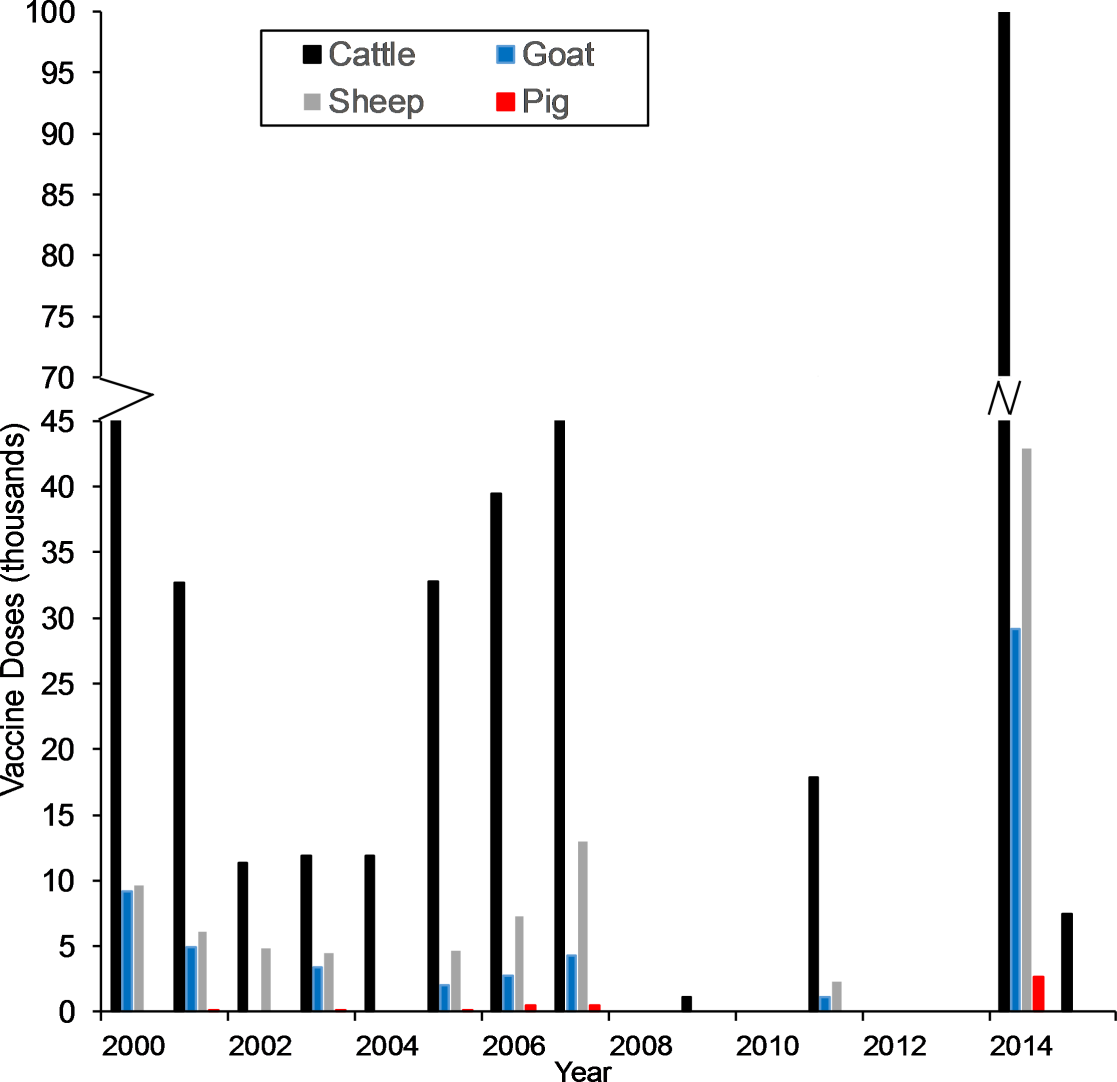
Annual livestock anthrax vaccine doses administered in Ghana during January 2005- June 2016.

Trends in livestock anthrax vaccination among livestock type are shown in Fig 4. From 2000–20015, there was a median of 17,957 doses [0–175 thousand] of anthrax livestock vaccine administered annually livestock vaccination occurred annually with a median number of doses administered of 19,709 [range: 0–175 thousand doses], followed by a decline in vaccine administration during 2008–2015. No vaccination was administered during the years 2010, 2012, and 2013. During 2008–2015, there was a median of 542 [range: 0–147 thousand doses] doses administered. In response to ongoing outbreaks, there was a vaccination campaign in 2014 that resulted in nearly an 8-fold increase in the number of doses administered compared to the previous six years. Among livestock types, cattle were most frequently administered vaccine, followed by sheep, goats and swine (Fig 4).

### Environmental suitability of anthrax in Ghana

**Fig 5:**
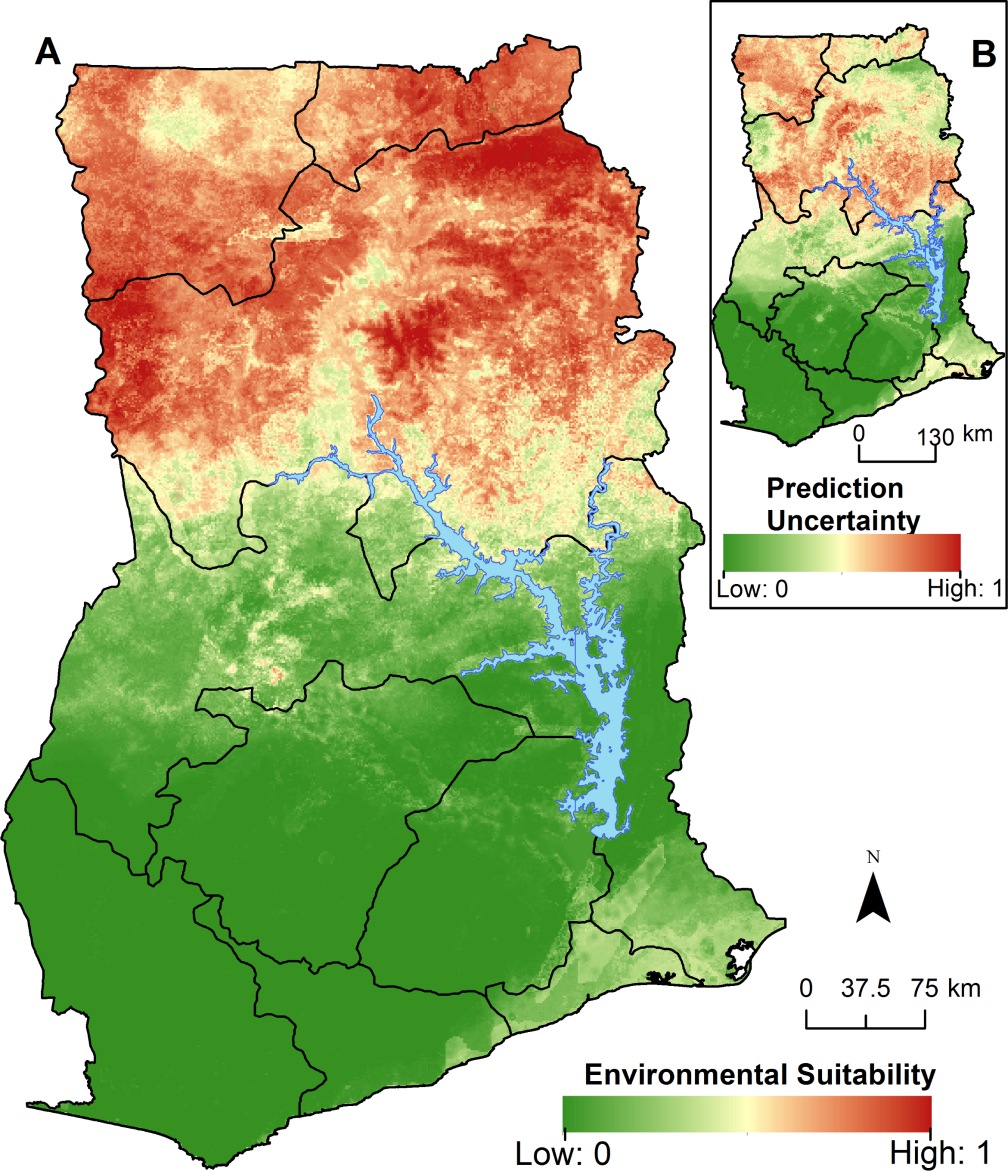
Environmental suitability of anthrax in Ghana as predicted by the ensemble random forest model (inset A). Uncertainty was calculated as the range of the 95% confidence intervals in predicted probability of suitability for each pixel, with areas of highest uncertainty in red, with greener colors representing low uncertainty (inset B).

The ensemble RF model suggests a latitudinal gradient in the environmental suitability of anthrax in Ghana (Fig 5A). High environmental suitability was identified in the Northern, Upper East, and Upper West regions of Ghana that encompass seasonal livestock migration routes from Burkina Faso in the north. Conversely, low or no environmental suitability was identified in southern Ghana among the more acidic soils in the Western, Ashanti, Central, and Eastern regions. Uncertainty (range: 0–0.20) in the model prediction was scaled from 0 to 1 and showed it was highest in the Upper West and Northern regions (Fig 5B). The internal OOB model validation indicated excellent discrimination with an AUC = 0.88 (95% CI: 0.87, 0.89). The external validation of anthrax outbreak locations withheld from the model (testing data) also showed excellent discrimination (AUC = 0.87 [95% CI: 0.85, 0.90]).

**Fig 6:**
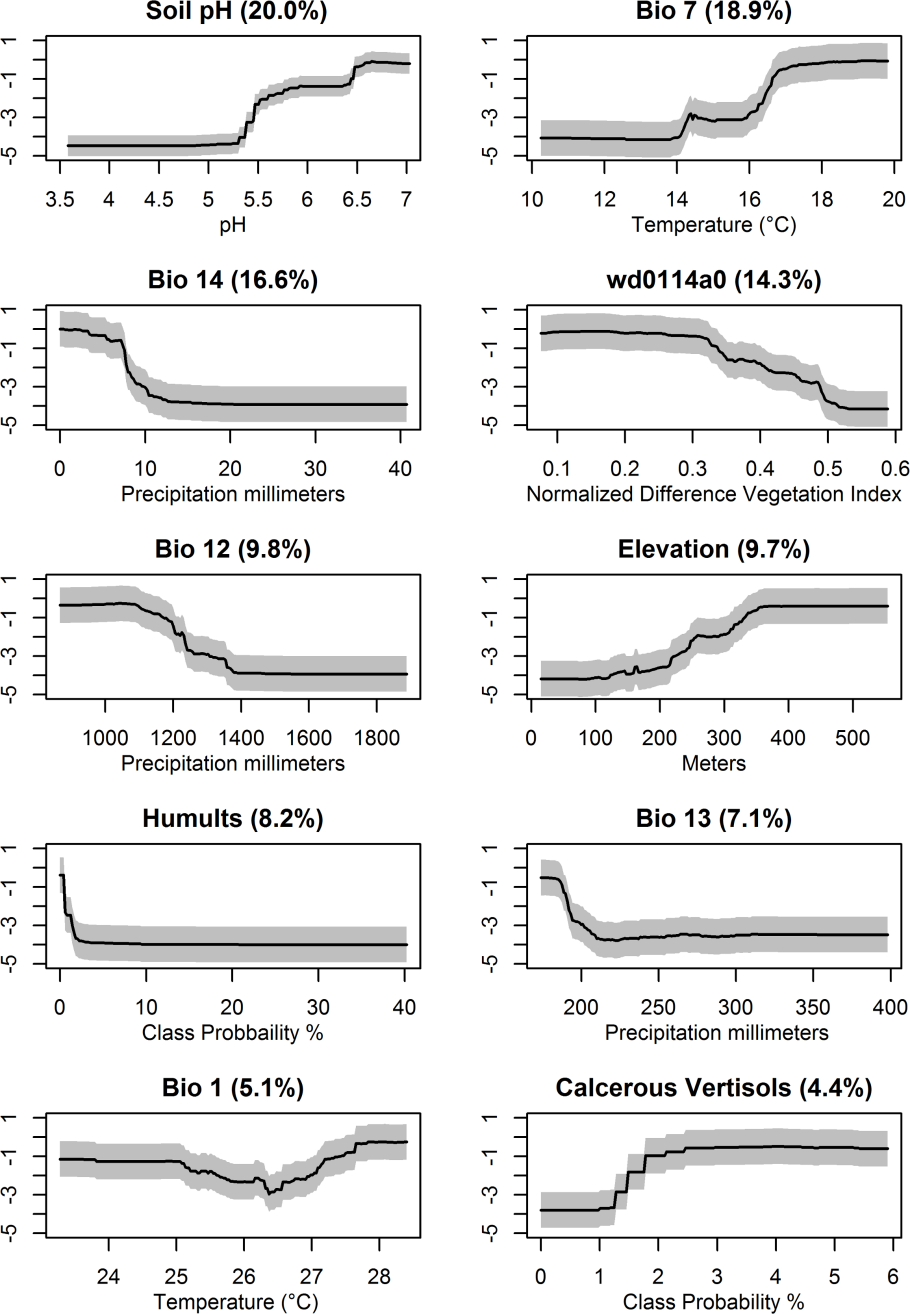
Partial dependency plots of environmental variables used in the random forest. Gray shading represented confidence intervals derived from the model iterations.

The final list of variables used in the ensemble model are shown in Fig 6. A combination of bioclimatic, environmental, and soil characteristics had the greatest impact on the OOB prediction errors. The most important variables influencing accuracy were: soil pH, bio7 (annual temperature range), and bio14 (precipitation of the driest month) (S2 Fig). The probability of the occurrence of anthrax increased in a step like manner in response to soil pH, increasing as the soil became more alkaline, between 5.5 and 6.5, and again between 6.5 and 7.0. Annual temperature ranges between 16 and 20°C were also related to a greater probability of occurrence. The occurrence of anthrax showed an affinity for low values of precipitation during the driest month (0 to 10 mm) and then dropped off dramatically as precipitation increased from 10 to 40 mm. Furthermore, as average NDVI (wd0114a0) increased from 0.3 to 0.6 the probability of anthrax occurrence decreased linearly, with a more suitable range of vegetation greenness identified in the lower ranges between 0.1 and 0.3 (Fig 6).

### Estimating populations at risk

**Fig 7:**
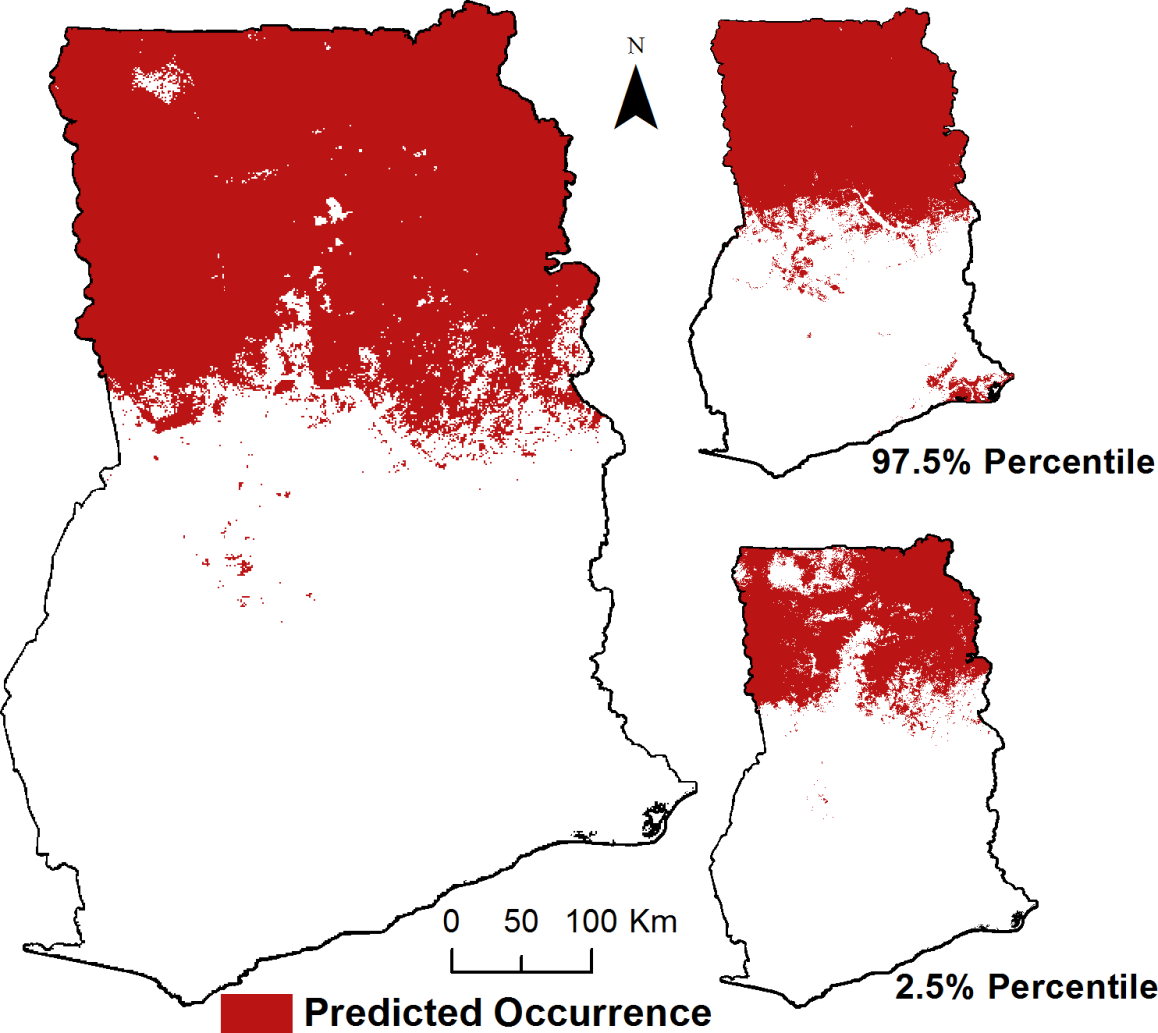
Dichotomized prediction of anthrax suitability with lower (2.5%) and upper (97.5%) occurrence limits. Dichotomized prediction was calculated using an optimal probability threshold (0.46) that maximized sensitivity plus specificity.

To estimate livestock and human populations at risk, we dichotomized the environmental suitability prediction (on a continuous probability scale) into suitable versus non-suitable environments for anthrax based on the optimal threshold (0.46) that maximized sensitivity (0.78) plus specificity (0.89) (Fig 7). The dichotomized prediction shows a marked north-south demarcation in the suitability of anthrax, with a majority of northern Ghana predicted as suitable within the accompanying upper (97.5%) and lower (2.5%) confidence bounds.

The national livestock population located in areas environmentally suitable for anthrax was estimated to be ≈ 2.2 (95% CI: 2.0, 2.5) million (Table 3). More than 50% of the livestock populations at risk were sheep and cattle (650 [95% CI: 583, 745] thousand and 480 [95% CI: 434, 527] thousand, respectively). Among livestock production systems, semi-arid rain-fed, mixed crop livestock systems (MRA) contained the greatest number of livestock at risk > 1.2 (95% CI: 1.1, 1.3) million (Table 3).

**Table 3:** Livestock production systems^¥^ and the livestock population in the Ghanaian anthrax risk zone.

	Population at risk [95% CI] (thousands of livestock)				
Livestock	LGA	LGH	MRA	MRH	**MIA**
cattle	108.5 [83.1, 123.2]	38.2 [30, 48.8]	283.3 [272.3, 289.7]	49.2 [47.7, 63.8]	1.2 [1.1, 1.2]
sheep	166.2 [125.4, 188]	50.6 [41.2, 74]	368 [353.8, 376.6]	63.9 [61.6, 105.5]	1.2 [1.1, 1.2]
goats	262.3 [201, 301.7]	47.9 [38, 67.8]	509.9 [491.3, 524.2]	117.8 [115, 164]	1.1 [1.0, 1.1]
swine	32.1 [24.7, 36.1]	11.5 [9.8, 17.1]	70.6 [67.6, 72.2]	14.7 [13.9, 24.9]	0.2 [0.2, 0.3]

^¥^ Livestock production systems (http://www.fao.org/docrep/014/i2414e/i2414e.pdf)

LGA: Livestock only systems, arid and semi-arid

LGH: Livestock only systems, humid and sub-humid

MRA: Rainfed mixed crop/livestock system, arid and semi-arid

MRH: Rainfed mixed crop/livestock system, humid and sub-humid

MIA: Irrigated mixed crop/livestock systems, arid and semi-arid

Nationally, there are approximately 3 million low income rural livestock keepers in Ghana (Table 4). Our model suggests that ≈ 805 (95% CI: 519, 890) thousand are located in areas suitable for anthrax, with the majority located in a humid and sub-humid, mixed crop livestock system production zone (MRH).

**Table 4:** Rural low income livestock keepers in the Ghanaian anthrax risk zone by livestock production system.

	Population at risk [95% CI] (thousands of people)				
Estimates	^¥^LGA	LGH	MRA	MRH	**MIA**
National	71.1	238.9	411.9	2152.9	1.2 [1.1, 1.2]
Modeled	48.6 [34.1, 56.9]	105.4 [65.7, 120.2]	300 [263.6, 325.1]	346.6 [155, 387.5]	0.4 [0.3, 0.4]

^¥^ Livestock production systems (http://www.fao.org/docrep/014/i2414e/i2414e.pdf)

LGA: Livestock only systems, arid and semi-arid

LGH: Livestock only systems, humid and sub-humid

MRA: Rainfed mixed crop/livestock system, arid and semi-arid

MRH: Rainfed mixed crop/livestock system, humid and sub-humid

MIA: Irrigated mixed crop/livestock systems, arid and semi-arid

## Discussion

Anthrax is a globally distributed neglected disease that is often underreported, particularly in West Africa where it is hyper-endemic [1,2,6,13]. Given the reliance of control on the vaccination of livestock, understanding the occurrence of anthrax is crucial for identifying populations at risk in order to disseminate limited resources. Here, we used data on the location of livestock outbreaks to identify seasonal patterns and model the environmental suitability of anthrax in Ghana. In keeping with previous studies, our findings indicate a defined outbreak season with a combination of ecological constraints on the potential geographic distribution of anthrax [3,34]. Our modeled prediction suggests a marked ecological divide separating the broad areas of environmental suitability in northern Ghana from the southern part of the country. Additionally, we estimated that populations characteristically at high risk for anthrax, which included >3 million combined ruminant livestock and poor rural livestock keepers are situated within the predicted anthrax risk zone. Based on our estimates, current anthrax vaccination efforts cover only a fraction of the livestock potentially at risk. Hence, these findings can be used to better direct public health intervention strategies and inform surveillance.

Official reports of livestock anthrax in endemic areas often go undocumented for a number of reasons, including the inability or unwillingness to report, limited surveillance capacity, and a lack of local knowledge about the disease [1]. In Ghana, livestock cases are likely underreported due to the slaughter and consumption of sick or dead animals [8,35], consistent with findings in the Caucasus and elsewhere [1,6,36,37]. This practice is often undertaken as a means of recouping economic losses from livestock mortality as well as providing food and a readily available source of protein [1,8,35]. The livestock anthrax outbreak data we used in this study were concordant with data reported to OIE during the same time frame suggesting Veterinary Services in Ghana are compliant with international reporting requirements (http://www.oie.int/wahis_2/public/wahid.php/Wahidhome/Home).

Despite the close proximity to the equator, we identified marked seasonality in anthrax reporting; outbreaks increased during the onset of the rainy season from February through April. Similar patterns of anthrax outbreaks associated with the rainy-season have also been reported in Namibia [34]. One hypothesis suggests that there is greater soil consumption among ruminants during with the rainy season [34], although soil exposure during the dry season has also been hypothesized as a cause of anthrax outbreaks [1]. Regardless, these findings suggest vaccination of livestock could be carried out in Ghana ahead of the peak outbreak season (September–November).

Livestock anthrax control in Ghana follows a similar trend in many endemic regions of reactively vaccinating in response to anthrax outbreaks [1,38]. In Ghana, the livestock population we identified at risk comprises approximately ≈ 25% of the total national livestock population [29]. Based on official vaccination reports (Fig 4), our estimates of the livestock populations at risk indicates poor vaccine coverage; this finding is consistent with ongoing outbreaks in endemic communities in Ghana where vaccination has not been officially documented for at least a decade [39]. Barriers to vaccine uptake such as practices of livestock keepers my also affect coverage [1,40]. However, Ghana faces additional control challenges with the potential presence of *B*. *cereus* biovar (bv) *anthracis* and West Africa strains (D and E Clades, respectively [41]). The West African strains have been hypothesized to evade the Sterne vaccine, which is the vaccine used in Ghana and throughout much of the world [13,42]. Further research is needed on vaccine efficacy and to understand what proportion of anthrax outbreaks are due to either insufficient application methods or the vaccine itself.

Research has suggested that soil pH >6.1 in conjunction with high calcium levels are a crucial component of *B*. *anthracis* spore survival [1,4,43]. Alkaline soils were also found to be associated with the persistence of anthrax transmission over several years [43,44]. In keeping with these findings, we identified an increasingly higher likelihood of anthrax occurrence in soils as pH increased from 5.5 to 7.0 and with an increasing level of calcareous vertisols. The association of anthrax suitability with lower levels of precipitation in our model is in line with reports that have documented soil nutrient leaching in regions with high precipitation, which may lead to soil acidification [45]. We predicted an area of environmental suitability for anthrax that encompasses ≈ 36% of Ghana’s total area (Fig 7); this is demarcated by a south (largely unsuitable) to north (highly suitable) divide, which closely mirrors the ecotone transitions from southern tropical and deciduous forests to the northern Sudanian and Guinea Savanna.

Our study had several limitations. As with all neglected zoonoses, our data likely represent an underestimation of the true burden of disease due to underreporting and limited resources for surveillance and testing. To better address issues with diagnostic testing and reporting we used a more contemporary dataset of anthrax outbreaks recorded during the last decade. Anthrax can also be transmitted from contaminated feed that is imported, and animal mortality may occur from livestock moved across long distances; however, we had no information on any outbreaks arising in these instances [1,46]. The use of machine learning algorithms to model the distribution of environmental pathogens has been well described, but such approaches, by their definition in conjunction with the use of averaged climate data, may over-generalize the landscape that supports the occurrence of anthrax outbreaks. Other factors not included in our models that may influence the occurrence of anthrax include the health and immune status of the livestock [47].

In conclusion, the current anthrax situation in West Africa, and in particular Ghana, remains a public and veterinary health threat due to challenges with reporting, surveillance, and control. Our findings suggest that broad areas of northern Ghana are environmentally suitable for anthrax. Furthermore, based on recent vaccination efforts, our estimates indicate that only a fraction of livestock at risk are being vaccinated. These findings can be used to help improve differential diagnostics, vaccine coverage estimates, and surveillance efforts. Given the reliance on agriculture and the large population of low income rural livestock keepers at risk in the northern part of the country where predicted suitability was highest, future control efforts should focus on improving livestock vaccination coverage and public awareness of the disease, prioritizing communities in the predicted anthrax zone.

## Supporting information

S1 Fig The spatial distribution of environmental variables used in the random forest models. Variable names are matched to variable descriptions and sources from Table 1.
(TIF)

S2 Fig Importance plot of variables included in the final ensemble random forest model prediction. Bars in darker blue represent variables that were more important in discriminating class prediction.
(TIF)
