# Supplementary figures and images for: Modeling the environmental suitability of anthrax in Ghana and estimating populations at risk: Implications for vaccination and control
Source: PLoS Negl Trop Dis. 2017 Oct 13;11(10):e0005885. doi: 10.1371/journal.pntd.0005885 (PMC5656412; doi:10.1371/journal.pntd.0005885)

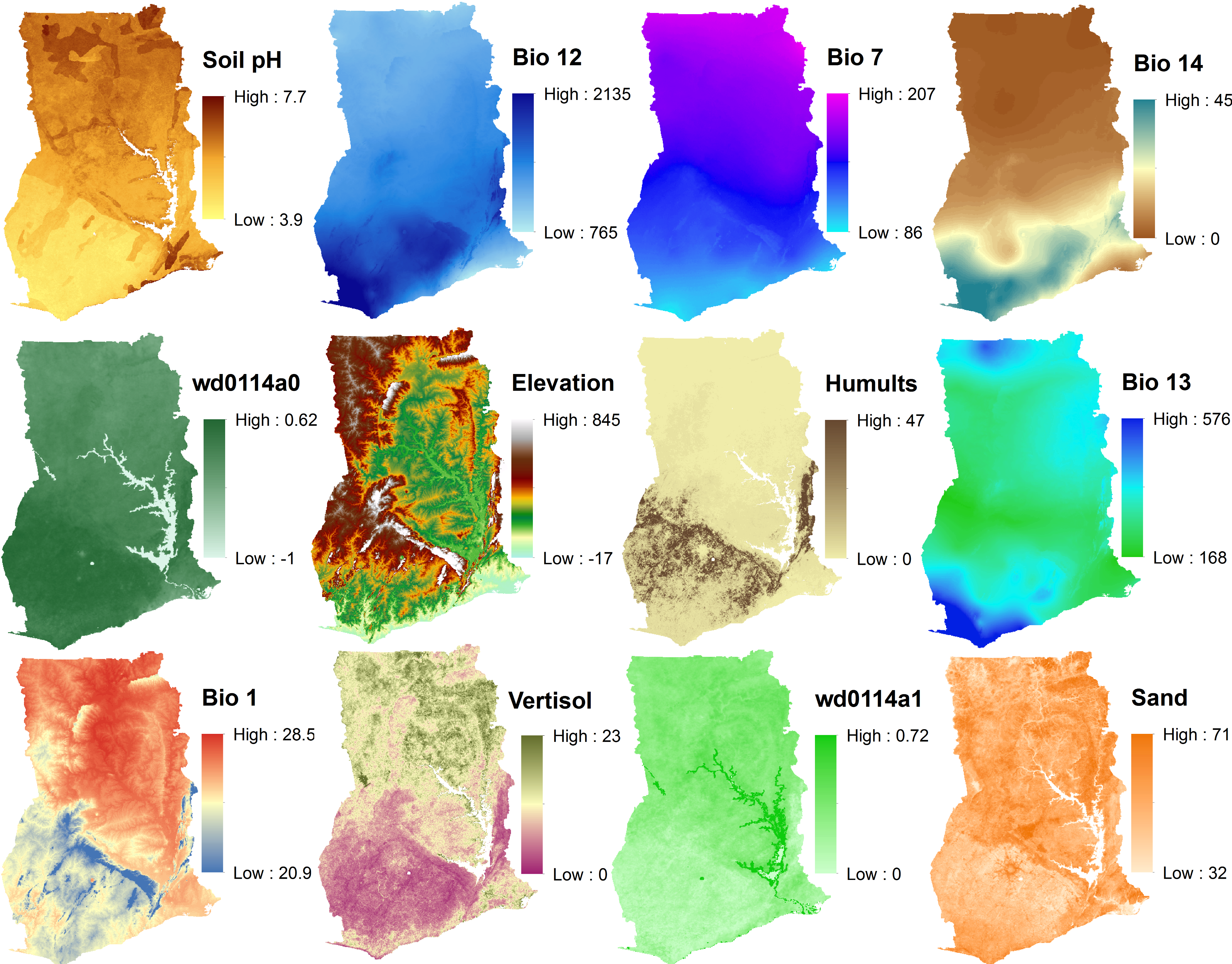

Supplement: S1 Fig — Variable names are matched to variable descriptions and sources from Table 1. (TIF) [file pntd.0005885.s001.tif]

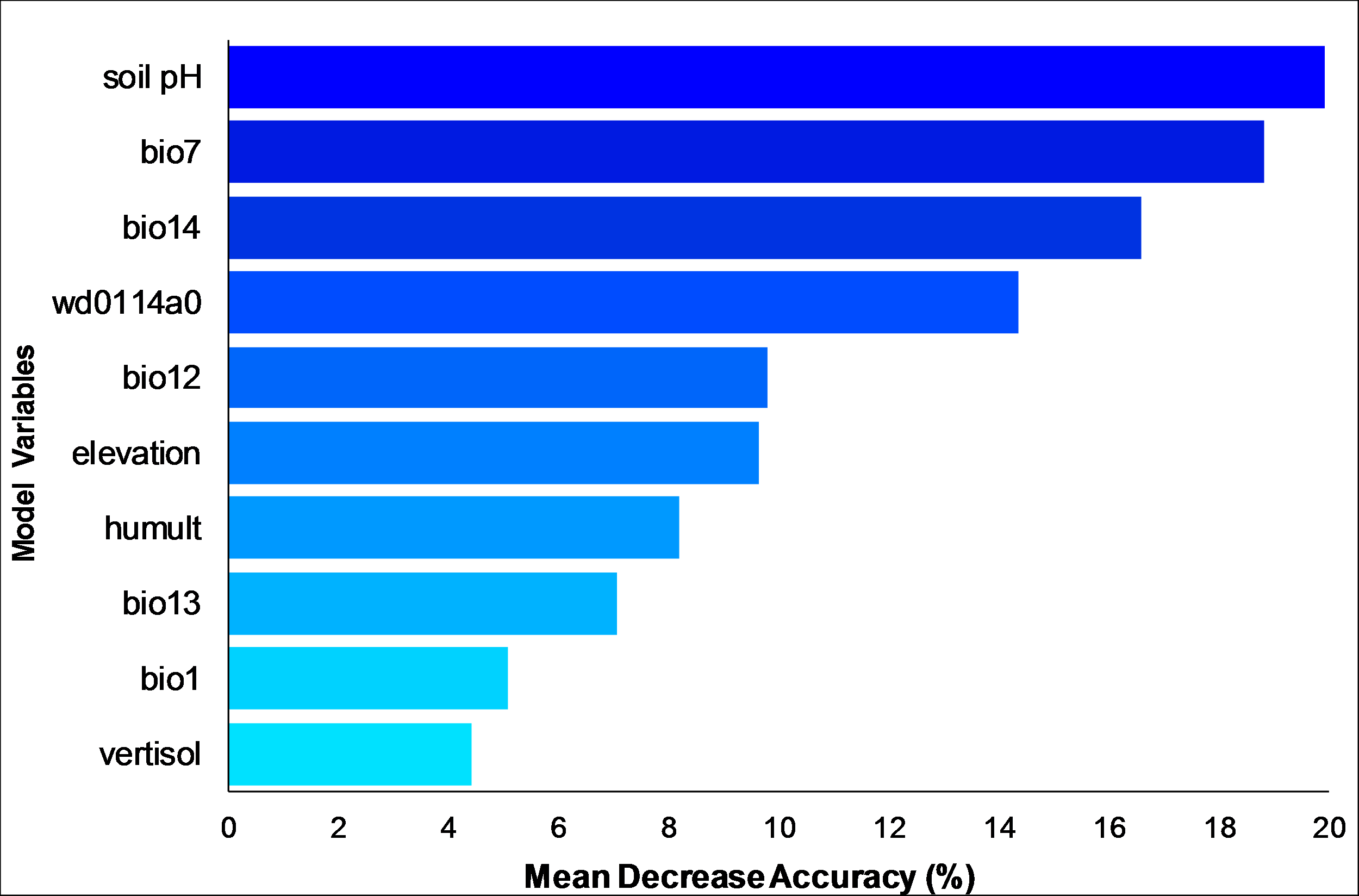

Supplement: S2 Fig — Bars in darker blue represent variables that were more important in discriminating class prediction. (TIF) [file pntd.0005885.s002.tif]
